# Radiographic outcomes of dorsal spanning plate with and without volar plate fixation for comminuted intraarticular distal radius fractures

**DOI:** 10.1016/j.jcot.2025.102949

**Published:** 2025-02-16

**Authors:** Daniel Calem, Matthew Weintraub, Michael Vosbikian, Irfan Ahmed

**Affiliations:** Rutgers Health New Jersey Medical School, Department of Orthopaedic Surgery, 140 Bergen St, ACC D1610, Newark, NJ 07103, USA

**Keywords:** Dorsal bridge plate, Radiographic outcomes, Wrist-spanning plate

## Abstract

**Purpose:**

Dorsal spanning plate fixation for comminuted intra-articular distal radius fractures involves indirect reduction via ligamentotaxis, potentially resulting in suboptimal restoration of native anatomy. Displaced volar fragments may necessitate separate buttress support with a volar plate. This objective of this study was to retrospectively compare radiographic outcomes between distal radius fractures managed with dorsal spanning plate fixation alone versus dorsal spanning plate fixation with concomitant volar plating.

**Methods:**

A retrospective review identified 51 distal radius fracture cases treated with dorsal spanning plate fixation, with 35 receiving isolated dorsal spanning plate fixation and 16 receiving dorsal spanning plate fixation with a concomitant volar plate. Radiographic parameters were measured at plate application and removal.

**Results:**

Final radiographs for isolated dorsal spanning plate fixation vs. dorsal spanning and volar plate fixation showed similar outcomes: radial height (9.3 mm vs. 8.9 mm, p = 0.8), ulnar variance (−2.77 mm vs. −2.47 mm, p = 0.76), radial inclination (18.8° vs. 16.3°, p = 0.21), volar tilt (1.3° vs. 2.8°, p = 0.45), and teardrop angle (48.9° vs. 51.0°, p = 0.32). Little to no loss of radiographic alignment was observed between time points.

**Conclusions:**

Radiographic outcomes for distal radius fractures treated with dorsal spanning plate fixation alone versus dorsal spanning plate and volar plate fixation are comparable, with suboptimal restoration of volar tilt and teardrop angle.

**Level of evidence:**

Therapeutic IV.

## 1 Introduction

**Fig. 1:**
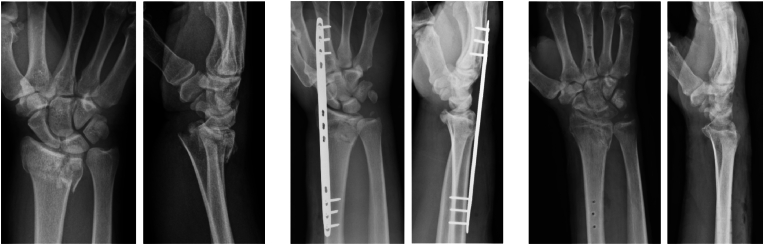
AP and lateral views for patient in DSP group at initial injury, after DSP fixation, and after DSP removal.

**Fig. 2:**
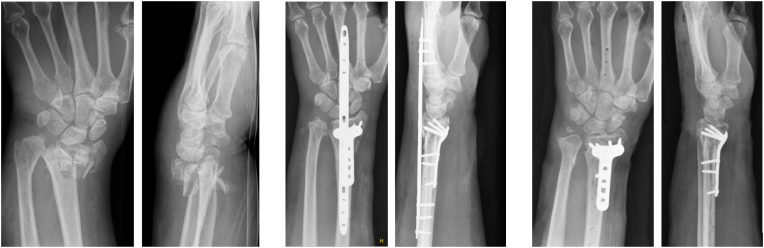
AP and lateral views for patient in the DSP + VP group at initial injury, after DSP and volar plate fixation, and after DSP removal.

Multifragmentary fractures of the distal radius with intraarticular and metaphyseal comminution are difficult injuries to manage. Limited bone stock may preclude stable fixation with solely a volar locking plate. The use of a dorsal spanning plate (DSP) offers an alternative treatment option in lieu of external fixation. DSP fixation involves the indirect reduction of intra-articular fracture fragments via a longitudinal distraction force and subsequent ligamentotaxis, and thus functions as an “internal ex fix.”^1^ This method has the potential advantages of a lower rate of infection and complex regional pain syndrome, increased construct stability sufficient to allow early digital motion and light functional use, and a more aesthetically appearing wrist compared to external fixation.^2^, ^3^, ^4^, ^5^ Disadvantages include the need for an additional surgical procedure for plate removal, as well as less direct control of fracture fragments and thus decreased ability to restore native anatomy (see (Fig. 1, Fig. 2).

During distal radius fracture fixation, restoring volar tilt and ulnar variance are vital in ensuring an optimal functional result.^6^ Loss of volar tilt and teardrop angle have been shown to be significant risk factors for increased pressure on the flexor pollicis longus (FPL) tendon and postoperative flexor tendon rupture.^7^^,^^8^ While some studies have reported adequate overall radiographic results and volar tilt restoration following DSP fixation,^9^, ^10^, ^11^ others have reported inadequate restoration of radiographic parameters,^12^ namely volar tilt and teardrop angle.^1^^,^^13^

The use of adjuvant surgical cointerventions, such as K-wire fixation, fragment specific fixation, and volar plating may better restore native anatomy. One recent study^14^ examined radiographic outcomes in patients with complex intra-articular distal radius fractures treated with DSP augmented with K-wire fixation, and reported improvement in these radiographic parameters compared with DSP alone. However, no studies have been performed comparing distal radius fractures treated with DSP in isolation compared with fractures treated with both a DSP and volar plate.

The objective of this study was to retrospectively compare radiographic parameters of distal radius fractures that were surgically treated with DSP in isolation with fractures that were treated with both a DSP and volar plate. The selected analysis focused on radiographic outcomes, as patient reported outcomes were not widely available in the study cohort. We hypothesized that these parameters, including radial height, radial inclination, ulnar variance, volar tilt, teardrop angle would be similar when comparing patients who had undergone DSP fixation alone or DSP with concomitant volar plating.

## 2 Methods

Institutional review board approval was obtained for this study. A retrospective review identified 515 wrists that underwent surgical treatment for a distal radius fracture over a ten-year period from July 2013 to July 2023 using current procedural terminology code search (25608, 25609). A retrospective cohort study design comparing DSP fixation alone (DSP group) with DSP and concomitant volar plate fixation (DSP + VP) was planned. Patients were included if they were 18 years or older, had a multifragmentary intraarticular distal radius fracture (AO type 23-C3), if surgical treatment included a DSP, and if removal of the DSP was performed. Patients lost to follow-up, those with no documented plate removal or radiographs following plate removal, those treated with DSP for other injury patterns (including radiocarpal dislocation), and pathologic fractures were excluded from analysis.

Medical records and imaging were manually reviewed to determine method of fixation. Pre-operative radiographs and computed tomography imaging were reviewed to determine fracture morphology. Of the 515 wrists that underwent fixation for a distal radius fracture, 65 were classified as AO type 23-C3 and managed with a DSP. Six patients were lost to follow-up and did not have documented DSP removal and were thus excluded from analysis. Eight did undergo DSP removal but did not have any postoperative radiographs or fluoroscopic images saved and were also excluded. This left 51 wrists available for analysis. All surgeries were performed by 1 of 6 surgeons fellowship-trained in either hand or orthopaedic traumatology.

Indications for DSP fixation included distal radius fractures with significant intra-articular comminution and/or depression, distal fractures extending into the volar or dorsal rims, and comminuted fractures with radiocarpal instability. The decision to employ the DSP technique and the decision to use the second or third metacarpal as the distal fixation point were not standardized and were at the discretion of the treating surgeon. Concomitant volar plating was employed for fracture management as dictated by fracture pattern and surgeon discretion, as were additional fixation methods including radial styloid pins or screws, suture anchors, distal radioulnar joint (DRUJ) pins, tension bands, ulnar styloid pins, and fragment specific fixation (Table 1). In terms of fixation sequence in cases of concomitant volar and dorsal spanning plate fixation, a volar approach was conducted first, followed by fracture fragment reduction and volar fixation. If additional stability or reduction was required, a dorsal approach was then made, dorsal reduction maneuvers were performed if required, and a DSP was applied. Patients with acute carpal tunnel syndrome or with fracture patterns that otherwise threatened the median nerve underwent carpal tunnel release during volar approach. DSPs were removed at the time of radiographic union. Volar plates were not removed routinely. Patient information including demographics, comorbidities, mechanism of injury, fracture characteristics, associated injuries, duration of follow-up, operative techniques, and complications were recorded.

**Table 1:** Demographic Data Comparing DSP vs. DSP + VP Groups.

'	DSP (N = 35)			DSP + VP (N = 16)			
	Value	Range	SD or %	Value	Range	SD or %	P-value
Age at Injury	43.0	18–83	19.5	41.4	21–74	16.3	0.777
BMI	27.0	15–37.9	4.0	25.8	0–35.4	7.8	0.482
Days between DOI and DOS	3.6	0–18	4.0	5.9	0–36	9.4	0.218
Weeks between DOA and DOR	23.2	6.0–204.6	32.6	20.2	12.3–81.1	16.6	0.734
Male	24		68.6 %	11		68.8 %	0.990
Smoker	10		28.6 %	5		31.3 %	0.846
Left wrist	21		60.0 %	11		68.8 %	0.549
2nd metacarpal	19		54.3 %	5		31.3 %	0.126
3rd metacarpal	16		45.7 %	11		68.8 %	0.126
Open	7		20.0 %	3		18.8 %	0.917
CTR	10		28.6 %	9		56.3 %	0.058
**Mechanism**							
Ground level fall	8		22.9 %	5		31.3 %	0.523
Fall down stairs	2		5.7 %	1		6.3 %	0.940
Fall from height	10		28.6 %	3		18.8 %	0.455
MVC	10		28.6 %	5		31.3 %	0.846
Pedestrian struck	3		8.6 %	1		6.3 %	0.775
GSW	1		2.9 %	0		0.0 %	0.495
Crush injury	1		2.9 %	1		6.3 %	0.562
**Additional Wrist Injuries**							
Distal ulna injury	11		31.4 %	2		12.5 %	0.150
DRUJ injury	5		14.3 %	3		18.8 %	0.684
Carpal injury	5		14.3 %	3		18.8 %	0.684
Finger injury	2		5.7 %	1		6.3 %	0.940
Acute CTS	4		11.4 %	6		37.5 %	0.030∗
**Additional Fixation**							
Radial styloid pin	12		34.3 %	6		37.5 %	0.824
Radial styloid screw	2		5.7 %	0		0.0 %	0.329
Suture anchors	4		11.4 %	2		12.5 %	0.912
DRUJ pin	5		14.3 %	3		18.8 %	0.684
Tension band	1		2.9 %	0		0.0 %	0.495
Ulnar styloid pin	3		8.6 %	0		0.0 %	0.227
Fragment specific plates	1		2.9 %	5		31.3 %	0.003∗

DOI: date of injury; DOS: date of surgery; DOA: date of DSP application; DOR: date of DSP removal; CTR: carpal tunnel release; MVC: motor vehicle collision; GSW: gunshot wound; CTS: carpal tunnel syndrome.

Radiographic parameters including radial height (mm), radial inclination (degrees), and ulnar variance (mm) were measured on the AP view and volar tilt (degrees) and teardrop angle (degrees) were measured on the lateral view. These parameters were measured digitally using PACS software using the methods described by Medoff.^15^ Radiographs were analyzed at two timepoints: after DSP application and after DSP removal. The radiographic data was measured by a single reviewer and reexamined by a second reviewer trained by an MSK radiologist.

Of note, 9 patients did not have postoperative radiographs at the time of plate removal; however, 8 of these had AP and lateral intraoperative fluoroscopic images and 1 patient had a lateral fluoroscopic image recorded; these were included in analysis. For these patients, radiographic parameters that included angulation could still be measured (radial inclination, volar tilt, teardrop angle), but not parameters that included length, as the fluoroscopic radiographs were not calibrated for length. However, angles were measured in the same manner as previously described for standard post-operative radiographs. Prior studies were used to define acceptable parameters as radial height >8 mm, radial inclination >15°, ulnar variance −3 > x > 3 mm, and volar tilt > −5°.^12^^,^^14^^,^^16^^,^^17^

The demographic characteristics of the 2 cohorts were reported as mean and SD for continuous variables and number and frequency for categorical variables. All radiographic data were reported as a mean and SD. Paired t-tests were used to compare continuous variables. Chi-squared tests were used to compare categorical variables. All tests were two-sided. A value of p < 0.05 was considered statistically significant.

## 3 Results

The final cohort included 51 patients with distal radius fractures treated with DSP fixation. The DSP group included 35 fractures (69 %) and the DSP + VP group included 16 fractures (31 %). Demographic data for both groups are included in Table 1. The mean ages were 43 (SD 19.5) and 41.1 (SD 16.3) for the DSP and DSP + VP groups respectively. The DSP group underwent fixation on an average of 3.6 days from injury (SD 4.0) and the DSP + VP group underwent surgery at an average of 5.9 days from surgery (SD 9.4); this difference was not statistically significant. In the DSP group, patients underwent plate removal an average of 23 weeks after plate application (SD 32.6) and in the DSP + VP group, patients underwent plate removal 20.2 weeks after plate application (SD 16.6). Other demographic variables including gender, smoking status, and laterality of extremity injured were similar between groups and not significant. Seven of the 35 fractures in the DSP group (20 %) were classified as open, and 3 of the 16 in the DSP + VP group (18.8 %) were classified as open. Nineteen DSPs (54.3 %) and 5 DSPs (31.3 %) in the DSP and DSP + VP groups were placed on the second vs. third metacarpal. This difference was not significant.

Associated hand/wrist injuries are also depicted in Table 1. Of note, 4 patients (11.4 %) were diagnosed with acute carpal tunnel syndrome in the DSP group compared with 6 patients (37.5 %) in the DSP + VP group, which was statistically significant (p = 0.03). Additionally, rates of carpal tunnel release were higher in the DPS + VP group compared with the DSP group (56.3 % vs. 28.6 %). This difference approached statistical significance (p = 0.058). In addition to volar plating, other adjuvant fixation methods included were radial styloid pins vs, screws, suture anchors, DRUJ pins, tension bands, and ulnar styloid pins. Five patients (31.3 %) in the DSP + VP group underwent additional fragment specific plate fixation either ulnarly, radially, or dorsally compared with one patient (2.9 %) who had a lateral radial plate placed in the DSP group. This difference was significant (p = 0.003).

Radiographic parameters of all 51 patients in the cohort at the time of both plate application and removal are noted in Table 2. Data stratified between the two groups are shown in Table 3. There were no statistically significant differences in any of the 6 radiographic parameters between groups at either time point. Importantly, evaluation of the change in each parameter at DSP application vs. removal was minimal, suggesting minimal loss or change in radiographic alignment from the time of surgery to bony union. 12/29 (41 %) of wrists in the DSP group and 5/13 (38 %) of wrists in the DSP + VP group met criteria for acceptable ulnar variance between −3 and 3 mm at the time of plate removal. Regarding volar tilt, 30/35 (86 %) of wrists in the DSP met criteria of > -5° versus 12/16 (75 %) of wrists in the DSP + VP group. For radial height, 19/29 (66 %) vs. 6/13 (46 %) met criteria of >8 mm and for radial inclination, 25/34 (74 %) vs. 10/16 (63 %) met criteria of >15° for the DSP and DSP + VP groups respectively.

**Table 2:** Radiographic data for all patients (N = 51).

	DOA			DOR			Difference		P-value
	Mean	(Range)	SD	Mean	Range	SD	Mean	SD	
Radial Height (mm)	9.6	(2.9–18.7)	3.6	9.4	(2.2–16.3)	3.5	−0.7	2.8	0.24
Ulnar Variance (mm)	−3	(-9.1 - 8.3)	2.8	−2.7	(-7.7 - 6.3)	2.9	0.4	3	0.45
Radial Inclination (degrees)	18.73	(4.6–34.1)	6.78	18.03	(4.2–32.3)	6.55	−0.44	3.14	0.32
Volar Tilt (degrees)	0.94	(-10.7–14.2)	6.28	1.93	(-11.1–15.7)	6.51	0.81	3.74	0.13
Teardrop Angle (angle)	49.6	(32.1–67)	9.03	49.57	(34.3–66.1)	6.97	−0.04	8.3	0.97

**Table 3:** Radiographic Data Comparing DSP vs. DSP + VP Groups.

	DSP (N = 35)			DSP + VP (N = 16)			P-value
	Mean	(Range)	SD	Mean	(Range)	SD	
**DOA**							
Radial Height (mm)	9.85	(3.7–16.9)	3.34	9.08	(2.9–18.7)	4.25	0.49
Ulnar Variance (mm)	−2.82	(-9.1 - 8.3)	3.23	−3.54	(-6.6 to −1.4)	1.61	0.40
Radial Inclination (degrees)	19.24	(8–34.1)	6.56	17.60	(4.6–30.9)	7.32	0.43
Volar Tilt (degrees)	0.39	(-10.7–14.2)	6.48	2.13	(-6.7 - 13.8)	5.83	0.37
Teardrop Angle (degrees)	49.90	(32.1–67)	8.72	48.95	(34.1–63.3)	9.93	0.73
**DOR**							
Radial Height (mm)	9.25	(2.2–16.3)	3.35	8.94	(3.6–14.8)	3.88	0.80
Ulnar Variance (mm)	−2.77	(-7.70 - 6.30)	2.90	−2.47	(-2.47- 4.20)	2.87	0.76
Radial Inclination (degrees)	18.82	(4.2–32.3)	6.60	16.34	(5.4–26.9)	6.31	0.21
Volar Tilt (degrees)	1.28	(-11.1–12.6)	6.13	2.78	(-6.5 - 15.7)	7.38	0.45
Teardrop Angle (degrees)	48.91	(34.3–60.4)	6.49	51.01	(39.1–66.1.)	7.96	0.32
**Difference (DOR - DOA)**							
Radial Height (mm)	−0.93	(-9.9 - 2.9)	2.76	−0.11	(-8.3 - 4.3)	2.98	0.39
Ulnar Variance (mm)	0.04	(-7.6 - 8.7)	2.97	1.05	(-3.8 - 7.8)	3.12	0.32
Radial Inclination (degrees)	−0.06	(-5.3 - 5.6)	3.13	−1.26	(-7.1 - 4.4)	3.09	0.21
Volar Tilt (degrees)	0.89	(-6.6 - 12.7)	3.86	0.65	(-4.9 - 8.4)	3.60	0.84
Teardrop Angle (degrees)	−1.00	(-18.4–18.5)	7.70	2.06	(-18.2–17)	9.40	0.23

### 3.1 Complications

There were no postoperative infections documented. Three patients were noted to have fractured DSPs before plate removal: 2 patients in the DSP group and 1 patient in the DSP + VP group. One of these patients in the DSP group had a partial union and was treated with cancellous allograft at the time of plate removal. Lastly, one patient in the DSP + VP group developed significant heterotopic ossification and synostosis after initial fixation and underwent volar plate removal at the time of DSP plate removal. This was the only case of volar plate removal. This patient went on to develop severe posttraumatic arthritis with radiocarpal joint collapse, and eventually underwent wrist arthrodesis three years later.

## 4 Discussion

The dorsal spanning plate is a useful technique for distal radius fracture fixation in cases of significant intra-articular comminution, polytrauma, fracture dislocation, shear injuries, and geriatric patients with poor bone quality.^18^^,^^19^ This method not only allows fixation of these complex fractures, but also allows for weight-bearing through the wrist, an advantage in polytraumatized patients or geriatric patients who require ambulatory assistive devices. However, the DSP relies on indirect reduction of fracture fragments via ligamentotaxis which may result in suboptimal restoration of native anatomy. Intra-articular reduction may be adjusted via additional dorsal incisions centered over Listers tubercle, which can allow for the direct manipulation of fracture fragments, placement of subchondral bone grafts, repair of intercarpal ligament injuries, and augmentation of fracture fixation with K wires or additional dorsal plates.^18^ Displaced volar fragments including the critical volar ulnar corner, depression fractures of the lunate facet, and volar shear fractures may require a separate volar incision and buttress support through a fixed-angle volar plate.

Normal radiographic parameters of the wrist were described by Medoff and include radial inclination 23.6 ± 2.5°, radial height 11.6 ± 1.6 mm, ulnar variance −0.6 ± 0.9 mm, volar tilt 11.2 ± 4.6°, and teardrop angle of 70.7 ± 4.2°.^15^ Restoring ulnar variance and volar tilt have been described as the most important parameters for a successful functional result, and inadequately restoring volar tilt and teardrop angle increases risk for flexor tendon rupture.^6^, ^7^, ^8^ Radiographic results after DSP fixation in the literature are mixed. While some studies report an average volar tilt greater than 5° and near neutral ulnar variance,^9^, ^10^, ^11^ others report suboptimal radiographic parameters. Sharareh and Mitchell^12^ identified 24 non-elderly patients that underwent DSP fixation for comminuted intra-articular distal radius fractures and reported a mean radial height of 11.1 ± 3.7 mm, radial inclination of 19.7 ± 5.4°, ulnar variance 1.0 ± 2.4 mm, and volar tilt of only 1.4 ± 5.2°. Additionally, they noted that malalignment in at least one of these parameters was identified in 67 % patients at the time of union. Rambeau et al.^13^ retrospectively reviewed 23 patients with comminuted intra-articular distal radius fractures and noted a mean radial height of 11.3 ± 2.3 mm, radial inclination 20.2 ± 4.5°, and volar tilt of 6.3 ± 5.8°. However, teardrop angle at the time of plate removal remained 47.7 ± 8.3 and was not restored to a normal value.

Recently, Bradley et al.^1^ evaluated radiographic outcomes of DSP fixation to the second vs. third metacarpal, and found that radiographic outcomes between these two groups are similar with an average radial height on 9.3 mm, radial inclination 17.4°, ulnar variance of 0.2 mm. However, volar tilt remained inferior in both groups (second metacarpal 1.9 ± 6.6°; third metacarpal 1.7 ± 7.7°). The present study demonstrated similar radiographic findings, with an average radial height of 9.4 ± 3.5 mm, radial inclination of 18 ± 6.6°, and ulnar variance of −0.3 ± 0.3°. Similar to Bradley et al., volar tilt remained suboptimal at 1.9 ± 6.5°. Additionally, similar to Rambeau et al., teardrop angle also remained suboptimal at 49.6 ± 7°.

The use of adjuvant surgical cointerventions, such as K-wire fixation, fragment specific fixation, and volar plating may better reduce fracture fragments and restore native anatomy. Shah et al.^14^ examined radiographic outcomes and maintenance of reduction in 35 patients with complex intra-articular distal radius fractures treated with DSP augmented with K-wire fixation, and reported improvement in these radiographic parameters compared to the Sharareh and Mitchell study. This included a statistically significant increase in volar tilt to 5.5 ± 6.2°.

The present study compared isolated DSP fixation with DSP in conjunction with volar plate fixation. The principal finding in this study is that radiographic outcomes for DSP with concomitant volar plate fixation remain similar to DSP fixation alone. Both groups demonstrated similar radiographic outcomes to those previously reported. While average volar tilt was slightly improved for the DSP + VP group, this was not significant (2.8 ± 7.4° vs. 1.3 ± 6.1°; p = 0.45).

This study demonstrates that while DSP fixation offers an attractive means of fixation for complex intra-articular distal radius fractures, restoration of radiographic parameters (namely volar tilt) may be inferior to less complex fractures treated with standard volar plating alone. While the DSP relies on eventual removal after bony union, other adjuvant hardware, including volar and fragments specific plates, routinely remain left in situ. While no cases of iatrogenic flexor tendon rupture were reported in this study, a residual volar plate may become a risk factor for iatrogenic flexor tendon rupture if volar tilt and teardrop angle are not adequately restored, especially for plates placed at Soong grade 1 or 2.^7^^,^^8^ Particular attention should be paid to these radiographic parameters at the time of plate removal. It may be beneficial to remove the volar plate in addition to the DSP at the time of DSP removal, particularly in patients without sufficient volar tilt or teardrop angle.

There are several limitations to this study. First, the retrospective, nonrandomized nature of this study introduces selection bias. Second, this study relied on manual measurement of radiographic parameters, introducing the possibility of measurement error. Manual measurements depend on the observer's skill and subjective interpretation, which can lead to inconsistencies, even under ideal conditions. This potential for error may affect the accuracy of the results and the validity of the conclusions drawn. While Watson et al.^20^ reported high intra and inter-observer reliability for volar tilt, as well as medium to high intra and inter-observer reliability for radial height, radial inclination, and ulnar variance, measurement error remains a possibility. Third, surgical fixation methods were not standardized among the patient cohorts. The decision to supplement fixation with a volar plate, as well as other methods such as K wires, fragment specific fixation, or suture anchors were at the surgeon's discretion, introducing potential confounding variability. Additionally, nine patients did not have postoperative plain films, and measurements relied on intra-operative fluoroscopic images. As such, distances were not calibrated and could not be measured. Lastly, this study was limited by a small sample size and may be underpowered to detect differences between groups.

In conclusion, the present study demonstrates that radiographic outcomes for distal radius fractures treated with dorsal spanning plate fixation alone are comparable to those treated with a combined dorsal spanning plate and volar plate construct, particularly in the suboptimal restoration of volar tilt and teardrop angle. While some comminuted intra-articular distal radius fracture patterns may have displaced volar fragments that necessitate separate buttress support with a volar plate in addition to a dorsal spanning plate to maintain length, the combined construct does not reliably improve radiographic parameters following reduction compared to dorsal spanning plate fixation alone.

## Patient consent

Informed consent was obtained from all individual participants included in the study.

## Informed consent

Informed consent was obtained from all individual participants included in the study.

## Credit author statement

Daniel Calem MD – conceptualization, methodology, formal analysis, investigation, writing (original draft)

Matthew Weintraub MD – investigation, formal analysis.

Michael Vosbikian MD – conceptualization, writing (review and editing), supervision.

Irfan Ahmed MD – conceptualization, writing (review and editing), supervision.

## Ethics statement

Ethical approval was obtained from the Institutional Review Board of Rutgers University.

(Submission ID Pro20170000549, approved April 2, 2024, expiration 2/19/2025).

## Disclaimer

The authors have not received grant support or research funding and have no conflicts of interest to declare.

## Funding statement

The authors declare that no funds, grants, or other support were received during the preparation of this manuscript.

## Declaration of competing interest

The authors declare that they have no known competing financial interests or personal relationships that could have appeared to influence the work reported in this paper.
